# A Multiplexed Cell-Based Assay for the Identification of Modulators of Pre-Membrane Processing as a Target against Dengue Virus

**DOI:** 10.1177/1087057115571247

**Published:** 2015-06

**Authors:** Zachary D. Stolp, Cameron A. Smurthwaite, Connor Reed, Wesley Williams, Andre Dharmawan, Hakim Djaballah, Roland Wolkowicz

**Affiliations:** 1 Department of Biology, San Diego State University, San Diego, CA, USA; 2 Institut Pasteur Korea, Seoul, South Korea

**Keywords:** dengue virus, high-throughput screen, cell-based assay, multiplexing, prM processing

## Abstract

The DenV pre-membrane protein (prM) is a crucial chaperone for the viral envelope protein, preventing premature fusion with vesicles during viral export. prM molecules in immature particles are cleaved by host proteases, leading to mature fusogenic virions. Blockade of prM cleavage would restrict fusion and represents a novel druggable opportunity against DenV. We have thus established a cell-based platform to monitor prM processing that relies on an engineered two-tag scaffold that travels to the cell surface through the secretory pathway. The assay discriminates between a single cell-surface tag when prM is cleaved and two tags when it is not, as detected through fluorescent-coupled antibodies by flow cytometry. The assay, miniaturized into a 96-well plate format, was multiplexed with the HIV-1 envelope boundary, also cleaved in the same pathway. A pilot screen against 1280 compounds was executed, leading to the identification of a potential active and corroborating the robustness of our assay for large-scale screening. We describe for the first time a cell-based assay that monitors DenV prM processing within the classical secretory pathway, which was exploited to identify a potential novel drug against DenV.

## Introduction

Dengue virus (DenV) infects 50–100 million each year and currently has no vaccine or treatment. DenV is the cause of dengue fever (DF) and the more lethal dengue hemorrhagic fever (DHF) and dengue shock syndrome (DSS).^1^ Transmitted primarily through mosquito vectors, mainly *Aedes aegyptii* and *Aedes albopictus*, DenV infects 50–100 million individuals each year, with an estimated 500,000 cases of DHF and 22,000 deaths.^1–4^ Furthermore, the World Health Organization estimates that 40% of the world’s population is at risk of infection.^1–4^ Currently, treatment is limited to palliative care, and no specific treatment (vaccine or antivirals) exists to combat DenV.^1–5^

DenV has four distinct serotypes and is a member of the *Flavivirus* genus within the family of Flaviviridae, which includes other important human pathogens such as the hepatitis C virus, West Nile virus, Yellow fever virus, and Japanese encephalitis virus. DenV enters the host cell, primarily phagocytic immune cells and hepatocytes, through receptor-mediated endocytosis.^6,7^ On viral entry, the 10.7 kb DenV RNA genome acts as mature mRNA, and is translated into a single polyprotein that is embedded within the endoplasmic reticulum (ER) membrane.^6–8^ Both viral and host proteases subsequently process the viral polyprotein into mature and active forms, including both structural and nonstructural proteins.^6–8^

DenV and other Flaviviruses contain three structural proteins essential for the formation of viral progeny: capsid (C), pre-membrane (prM), and envelope (E).^9–13^ Viral assembly is highly coupled to the ER compartment. Immature virions bud into the ER lumen and acquire a host-derived membrane containing both prM and E viral proteins.^6–8,12,14^ Viral progeny then use the secretory pathway and are released from the infected host cells by exocytosis.^6,8–10^

During viral exit, immature virions exploit cellular enzymes, namely, Furin and/or other Furin-type proteases, for the cleavage of the membrane-associated prM protein.^6,9,10,15–17^ Host-protease processing of prM into the soluble pr and membrane-associated M is required to allow E to mediate fusion during viral entry in subsequent rounds of infection.^9,13,18,19^ Although some reports in the literature note that DenV particles containing noncleaved prM molecules can still enter macrophages and dendritic cells via antibody-dependent enhancement, prM processing is presumably still required to escape the endosome.^9,18–20^ Thus, the blockade of prM processing represents an intriguing novel drug target against DenV. PrM is cleaved by host enzymes, thus drugs targeting prM processing should be competitors rather than inhibitors, because the latter would be cytotoxic.

We have previously described an assay that relies on a two-tag system to monitor the processing of the human immunodeficiency virus (HIV) envelope (Env) protein.^21^ Here, we describe for the first time the adaptation of the assay for the monitoring of DenV prM processing. The assay uses the expression of an engineered scaffold containing a putative substrate, the DenV prM boundary, flanked by two epitope tags, FLAG and HA. The scaffold is engineered to travel through the secretory pathway, mimicking natural viral transport, and is eventually embedded within the cell surface. In such a way, if the substrate is not cleaved, the scaffold displays both epitope tags (FLAG and HA). Conversely, if the substrate is processed, a tag (FLAG) is released from the scaffold and only one epitope (HA) is displayed on the cell surface. Tag surface display, specifically FLAG, thus serves as a biosensor for substrate cleavage and can be detected through fluorescently coupled antibodies by classical flow cytometry.

The growing technological capabilities of better and faster instrumentation coupled to assays both in vitro and ex vivo have had a huge impact on high-throughput screening (HTS) platforms that have propelled scientific and drug discoveries. However, most putative drug hits fail at later stages of development and/or implementation, and few hits actually become leads. Improving assay and screening technologies will certainly help identify better hit–lead compounds, thus reducing the cost involved in subsequent stages of drug development. Many current high-throughput technologies use cell-based assays to provide a more natural context for drug–target interactions as well as to address cytotoxicity. In addition, many platforms seek to multiplex to analyze drugs against similar targets simultaneously, reducing cost, time, and material. However, few platforms exist for multiplexed screening applications and are often adapted to more biochemical approaches rather than live cell-based platforms.^22,23^ Our two-tag system can specifically couple the observed phenotype with a specific cell within a population, allowing for multiplexing in a robust and reliable manner.^21,24^ For the first time, we have developed a novel, high-throughput, multiplexed, cell-based platform that relies on an antibody staining-based approach that can be readily exploited to analyze living cells in the presence of peptide or chemical compound libraries. In addition, although platforms for the discovery of DenV antivirals have been established,^5,25–27^ no such platform exists that specifically targets prM maturation while mimicking not only a cellular context but also, more specifically, the cellular compartment where prM cleavage occurs.

Here, we present the adaptation of our robust two-tag system to DenV prM processing and its miniaturization to a 96-well plate format. We have then multiplexed the assay using retroviral fluorescent genetic barcoding to further enhance the capabilities of the screening platform.^24^ The well-established Furin substrate of HIV Env was exploited for that purpose, while serving also as control for cleavage specificity.^15,21,28–33^ A pilot screen of 1280 small molecules contained in the Prestwick Chemical Library (PCL) was conducted using our established multiplexed assay and revealed putative hits, demonstrating the high-throughput multiplexed capabilities of our novel cell-based assay for drug discovery against DenV.

## Materials and Methods

**Construction of Plasmids and Vectors:** Assay constructs and vectors were engineered as follows. The constructs pBluescript.FLAG-HIV-1ENV-wt-Lyt2 and pBluescript.FLAG-HIV-1ENV-mut-Lyt2 (arginine–serine mutation) were previously engineered and contain sequences amplified from the HXB2 strain of HIV-1 as substrate.^21^ To insert DenV prM into the assay backbone, the substrate was engineered using overlapping primers (containing HindIII and EcoR1 restriction sites) to amplify the DNA corresponding to the 20 amino acids comprising the prM cleavage site of DenV Type 2 New Guinea isolate, kindly provided by Ronaldo Mohana (Federal University of Rio de Janeiro, Rio de Janeiro, Brazil). Amplified fragments were subsequently digested with HindIII and EcoR1, and were ligated into pBluescript.FLAG-HIV-1ENV-wt-HA-Lyt2 cut with HindIII/EcoR1. To transfer the assay into retroviral vectors for mammalian expression and cell line engineering, pBluescript versions of the assay were transferred into pBMN.iresZeocin vectors using Xho1 and Not1 restriction sites.

**Cell Maintenance:** Human T-cell line SupT1 was obtained from the American Type Culture Collection (ATCC, Manassas, VA). Cells were maintained in complete RPMI 1640 media supplemented with 10% fetal bovine serum (Gemini Bio-Products, West Sacramento, CA), glutamine (2 mM), penicillin G (100 units/mL), and streptomycin (100 µg/mL). Phoenix GP and HEK293T cell lines (Nolan Lab, Stanford University, CA) were maintained in Dulbecco’s Modified Eagle’s Medium supplemented with 10% fetal bovine serum (Gemini Bio-Products), glutamine (2 mM), penicillin G (100 units/mL), and streptomycin (100 µg/mL).

**Antibodies and Reagents:** Anti-FLAG antibody was obtained from Sigma-Aldrich (St. Louis, MO). Anti-HA, anti-mouse IgG Alexa Fluor 488, anti-rabbit IgG Alexa Fluor 647, anti-mouse IgG Alexa Fluor 555, and anti-rabbit Alexa Fluor 488 were obtained from Cell Signaling (Beverly, MA). Decanoyl-RVKR-chloromethylketone (DCK) was obtained from Tocris Bioscience (Bristol, UK) and was dissolved in dimethyl sulfoxide (DMSO).

**Viral Production and Transduction:** For the production of Moloney murine leukemia virus (MLV)-based retrovirus, a 10 cm^2^ plate of Phoenix GP cells at 50% confluence was transfected with 3 µg of the packaging vector (pBMN plasmids) and 3 µg of a vector expressing the envelope glycoprotein of the vesicular stomatitis virus (pCI-VSVg) by mixing the plasmids in 125 µl of serum-free DMEM with 30 µg of polyethylenimine (PEI) (linear, MW 24000; Polysciences, Warrington, PA).

For MLV-based viral production, media (DMEM with 10% FCS, Pen-Strep, L-glutamine) were replaced 24 h post transfection, and viral supernatant was collected 48 h after transfection and filtered through 0.45-µ polytetrafluoroethylene filters (Pall Corporation, Port Washington, NY). The supernatant was used to spin-infect SupT1 cells (1 million/mL) in a 12-well plate format. Briefly, viral supernatant was mixed with polybrene (5 mg/mL final concentrations) and added to the cells, and the mixture was plated in a 6- or 12-well plate and spun at 1500×g, 32 °C for 80 min in a hanging bucket rotors centrifuge (Becton-Dickinson, East Rutherford, NJ). Twenty-four hours post infection, fresh media (RPMI) were added to cells.

**Flow Cytometry and Sorting:** Cells were pelleted and incubated with mouse anti-FLAG (Sigma-Aldrich) and rabbit anti-HA (Cell Signaling) at 1:400 dilution for 20 min and then washed with phosphate buffered saline (PBS). Cells were then incubated with anti-mouse Alexa Fluor 488 and anti-rabbit Alexa Fluor 647 (Cell Signaling) antibodies at 1:200 dilutions for 20 min and washed with PBS. For staining and analysis of 96-well plates, cells were incubated with anti-FLAG and anti-mouse Alexa Fluor 488 at 1:2500 dilutions. PBS was subsequently added to dilute excess antibody. Flow cytometry and sorting were performed on a BD FACSCanto with 488 nm and 633 nm lasers and/or FACSAria with 488 nm and 633 nm lasers. For FITC and APC detection, a 530/30 band pass filter preceded by a 502 long pass filter and a 660/20 band pass filter were used, respectively. Data were collected on FACSDiva 6.1.1 and analyzed using FlowJo 7.6.5.

**Preparation of Library and HTS:** The original PCL, provided in 384-well plates at 1 mM, was copied into 96-well plates at 100 µM (10 µl of the original library into 100 µl). The 96-well plate at 100 µM served as stock for further copies of the library. Ten microliters of the stock were transferred into 96-well plates for screening. 3.0×10^4^ cells in 90 µl were then transferred into each well with a MultiDrop 384 (Thermo Scientific, Waltham, MA) to bring the final concentration of chemicals to 10 µM in 100 µL growth media. Cells were incubated in the presence of chemicals for 48 h, and analyzed for cytotoxicity and auto-fluorescence before staining. Each plate included positive and negative controls: a negative unstained control, a solvent control (1% DMSO), a stained control with no chemical for background staining, and a positive staining control (HIV Env-wt in the presence of DCK). To each well, 150 µL of a stock mixture containing mouse anti-FLAG primary antibody, anti-mouse Alexa 488 secondary antibody, and PBS mixture was added. The stock mixture per 96-well plate consisted of 5 µL of each antibody in 15 mL of PBS. The BD FACSCanto to high-throughput sampler was used for acquisition and analyzed using FlowJo 7.6.5 and CytoBank.

**Dose Response and IC_50_ Determination:** An independent source of dry material was used for calculating dose response (Tocris Bioscience). DenV prM cells were incubated with increasing concentrations of Thiostrepton and stained for FLAG-FITC. Each concentration was conducted in triplicate. Data were analyzed by FlowJo 7.6.5 and GraphPad Prism. A nonlinear regression with a four-parameter variable slope was used to fit data points, generate a dose–response curve, and predict half-maximal inhibitory concentration (IC_50_).

## Results

### Adaptation of the Assay to DenV prM

**Figure 1.**
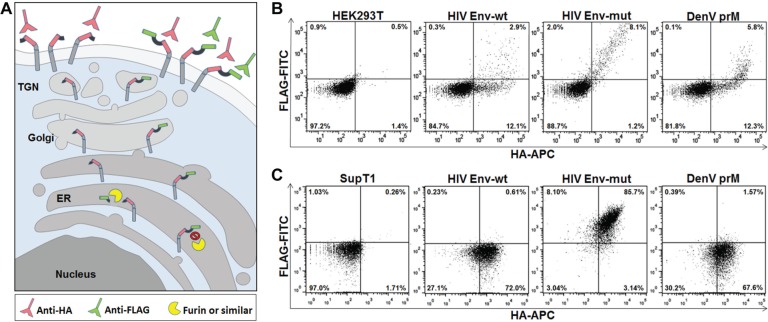
Cell-based assay for monitoring DenV pre-membrane protein (prM) processing. (**A**) Model of the assay. A scaffold protein carrying a substrate flanked by the HA and FLAG tags is engineered to travel to the cell surface through the classical secretory pathway. If the substrate is cleaved on its way to the surface (left scenario), it will be detected by only “red” antibodies. If noncleaved (right scenario), it will be recognized by both “red” and “green” antibodies. (**B**) Transfection experiment in HEK293T cells with the assay constructs harboring the DenV prM substrate, or the HIV Env-wt or mut substrates as controls. Cells were stained with anti-FLAG-FITC and anti-HA-APC, and analyzed by flow cytometry. (**C**) Retrovirally engineered SupT1 cell lines stably expressing the genetic information for each assay construct and stained as in **B**.

Our previously described assay to monitor HIV Env boundary processing within the ER trans-Golgi network was proven to discriminate between cleaved and noncleaved events in a very robust manner, and was thus the perfect platform for adaptation to DenV prM.^21^ The assay relies on the expression of a double-tagged (FLAG and HA) scaffold that carries the putative substrate, which travels to the cell surface through the secretory pathway. If the substrate is recognized and cleaved by endogenous proteases within the pathway, only the HA tag will be present on the cell surface.^21^ Conversely, if proteases fail to cleave the substrate, both epitopes will be presented on the cell surface (**Fig. 1A**). Thus, FLAG surface expression serves as a biological readout for substrate cleavage. The assay was engineered in a retroviral backbone vector for the development and selection of cell lines. Establishment of cell lines was necessary to decrease variability and thus improve readout conditions such as the signal-to-noise ratio and repeatability required for screening.

To adapt the existing two-tag assay to monitor DenV prM processing, 20 amino acids comprising the Furin (or similar) prM cleavage site from the type 2 New Guinea-C strain were introduced within the assay cassette in the retroviral vector pBMN, as described previously for the HIV-1 Env boundary.^21^ The newly engineered DenV prM construct was transfected into HEK293T cells alongside the previously engineered HIV Env constructs used as controls. Cells were subsequently stained with HA-APC to establish that the new scaffold traveled to the cell surface similarly to the control constructs. They were also stained with FLAG-FITC to assess whether cleavage occurred and thus corroborate assay functionality (**Fig. 1B**). It is worth noting that although transfection experiments and transient expression are not expected to prove the robustness of the assay, they are intended to prove that the anticipated trend is achieved. As expected, when cells were transfected with the wild-type HIV Env assay construct, a clear distinction between cell surface expression of cleaved (12.1% HA positive only) and noncleaved events (2.9% FLAG/HA positive) was observed. Furthermore, the noncleavable control HIV Env-mut, containing a single arginine-to-serine substitution (R511S) that abrogates cleavage, showed a robust noncleaved phenotype (8.1% FLAG/HA positive) (**Fig. 1B**). In comparison, cells transfected with the new DenV prM–containing assay construct exhibited a 12.3% single positive versus 5.8% double positive, indicating that the engineered DenV prM travelled to the surface and was cleaved, at least partially (**Fig. 1B**). The hook-like staining pattern for DenV indicates that whereas low expressors of the construct show cleavage, in high expressors the construct fails to be cleaved, indicating cleavage might not be efficient. Cells transfected with DenV prM are more resistant to cleavage as compared with HIV Env-wt (5.8% versus 2.9% FLAG/HA positive) (**Fig. 1B**), further corroborating reports in literature pointing out that DenV prM is known to be inefficiently cleaved.^9,10,19^ Importantly, HA staining of transfected cells clearly indicated that the engineered scaffold containing the DenV prM substrate functioned as expected.

On confirmation of assay functionality for monitoring cleavage of prM, we developed cell lines harboring the DenV prM assay using retroviral technology. SupT1 cells were chosen due to their non-adherent nature and easy analysis by flow cytometry. Moreover, although not a primary cell target of DenV infection, leukocytes have been shown to be permissive.^20,34^ Briefly, SupT1 cells were infected with retroviral particles harboring the genetic information for the assay. To produce clonal populations, single cells were sorted by FACS into 96-well plates and tested following amplification for assay expression as based on HA surface expression. Following further amplification, clonal populations were screened for FLAG and HA expression to ensure that the proper cell lines for assay detection were obtained. Chosen lines showed a robust expression profile when compared to their unstained controls. The HIV Env-mut cell line was double positive (85.7%) for HA and FLAG epitopes, whereas the HIV Env-wt remained single positive for HA (72%) (**Fig. 1C**). Similarly to HIV Env-wt, the DenV prM–expressing cells exhibited a single positive phenotype (67.6%) (**Fig. 1C**). Furthermore, genomic DNA isolated from the engineered cell line was verified and sequenced to eliminate the possibility that mutations occurred during retroviral insertion. Together, these results showed that we obtained a cell line that carried a scaffold protein adapted to the monitoring of DenV prM cleavage.

## Assay Miniaturization

Because the assay was ultimately engineered to facilitate HTS, it was important to miniaturize the assay to a 96-well plate format. The assay relies on detection (or lack of) of the FLAG epitope tag on the cell surface by fluorescent-coupled antibody staining and flow cytometry analysis. HTS platforms try to avoid antibody staining due to the cumbersome time-consuming staining protocols, sample preparation, and washing steps, which often result in the loss of sample cells. In an attempt to streamline sample preparation and assay analysis, as well as decrease the cost of antibodies for large-scale screens, the staining procedure was further calibrated to avoid washes. Washing of cells in staining protocols is often required to discard excess antibody and increase signal-to-noise ratios by decreasing nonspecific background staining.

**Figure 2.**
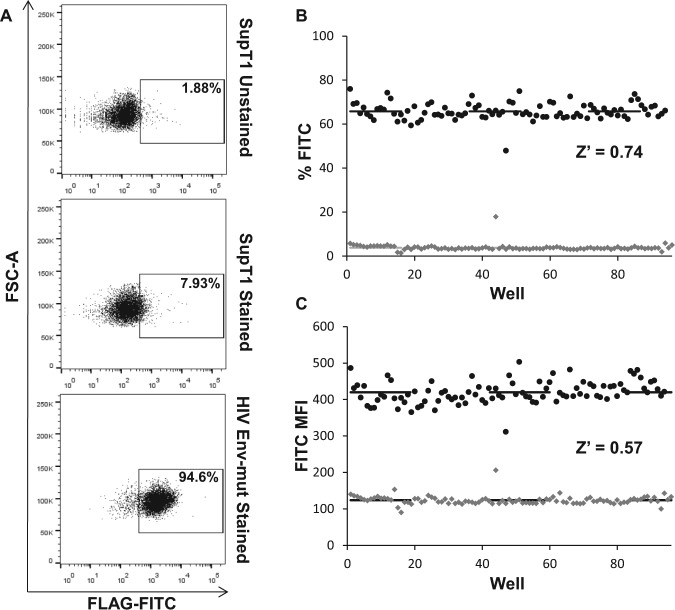
Assay miniaturization to 96-well plates. (**A**) Preliminary flow cytometry analysis of FLAG-FITC-stained SupT1 cells in 96-well plates with no wash. For this experiment, background and positive staining were performed with naïve and HIV-Env-mut cells. (**B, C**) Two 96-well plates harboring SupT1 Env-wt treated with DMSO (light gray bullets) or DCK inhibitor (dark gray bullets) were stained with FLAG-FITC and analyzed by flow cytometry to define the robustness of the assay. (**B**) Z’ analysis based on percent fluorescence. (**C**) Z’ analysis based on mean fluorescence intensity.

To miniaturize assay readout and improve high-throughput capabilities, we thus performed preliminary experiments to demonstrate the feasibility of a no-wash staining protocol. Briefly, naïve SupT1 cells as well as the HIV Env-mut cell line (as a positive staining control) were plated on 96-well plates and incubated for 48 h to simulate normal drug incubation times. Cells were then simultaneously incubated with anti-FLAG and anti-mouse conjugated to Alexa Fluor 488 antibodies diluted in PBS. Following staining, plates were directly analyzed by flow cytometry in the absence of washing. Whereas stained naïve SupT1 cells exhibited some expected nonspecific background staining (only around 7.93%), cells expressing the noncleavable form of the assay were strongly positive for FLAG (94.6%) (**Fig. 2A**), showing the assay can be readily analyzed in a miniaturized platform with a simplified, no-wash staining procedure.

We then addressed the reproducibility and repeatability of analysis to ensure that the 96-well plate format was suitable for HTS. Accordingly, 96-well plates containing SupT1 cell lines harboring the assay for monitoring HIV envelope processing (Env-wt cells) were analyzed using the miniaturized platform. The previously published and robust Env-wt cell line was chosen to establish nonbiased parameters for subsequent analysis of DenV prM. For that purpose, 96-well plates containing SupT1 Env-wt cells treated with DMSO solvent control or 50 µM of the known Furin inhibitor decanoyl-Arg-Val-Lys-Arg-chloromethyl-ketone (DCK) were stained for FLAG-FITC, without washing. DCK was shown to reconstitute FLAG surface expression because it inhibits Furin, the enzyme involved in HIV-1 Env cleavage. DCK is thus also used as a drug to further prove utility of the assay for drug screening. When plates were analyzed for percentage FITC staining (FLAG surface expression), cells treated with DCK were on average 65.79% positive with a standard deviation (σ) of 3.8% (**Fig. 2B**). However, cells treated with vehicle control yielded on average 3.85% ± 1.61 positive (**Fig. 2B**). To assess the robustness, repeatability, and reproducibility of assays, Z’ values between 0.5 and 1.0 are deemed excellent for HTS and analysis. When calculated for percentage fluorescence, the calibrated staining yielded a Z’ of 0.74 (**Fig. 2B**). Becuse percentage fluorescence is determined by arbitrary gates set against negative control cells, the data set was also analyzed by mean fluorescence intensity (MFI) to further demonstrate precision of the assay. When analyzed for MFI, the plate treated with DCK had a value of 419.65 with a 30.34 σ (**Fig. 2C**). Conversely, cells with DMSO vehicle control had a lower value of 123.76 ± 12.20 (**Fig. 2C**). When calculated based on MFI values, analysis of the calibrated staining yielded a Z’ value of 0.57 (**Fig. 2C**). These results demonstrated that the assay in a miniaturized form did exhibit a robust and repeatable readout that clearly distinguishes between positive and negative signals, making it suitable for HTS.

## Establishment of the Assay in a Multiplexed Format

Adaptation to the DenV prM substrate and calibration for HTS allowed us to further multiplex the assay with our assay constructs for HIV Env-wt and HIV Env-mut using fluorescent genetic barcoding as described previously.^21,24^ HIV-based constructs were thus used not only as controls for setting staining parameters but also for elucidating the specificity of potential hits encountered during the screening process. Multiplexing allows for the analysis of multiple experimental elements at the same time as used here for the different viral substrates. Coupling HTS with multiplexing can greatly enhance the discovery of putative drugs against different targets, while reducing cost and time. To multiplex the described assay, we have used the power of genetic engineering through retroviral technology and cell sorting to obtain cell lines with distinguishable fluorescent protein intensities, as described by Smurthwaite et al.^24^ Three distinct populations of cells expressing different intensities of td Tomato—negative, dim, and bright—were generated, each carrying a distinct construct (HIV Env-wt, HIV Env-mut, or DenV prM, respectively). The three cell lines can then be mixed in one sample and still be distinguishable based on their fluorescent barcode.

**Figure 3.**
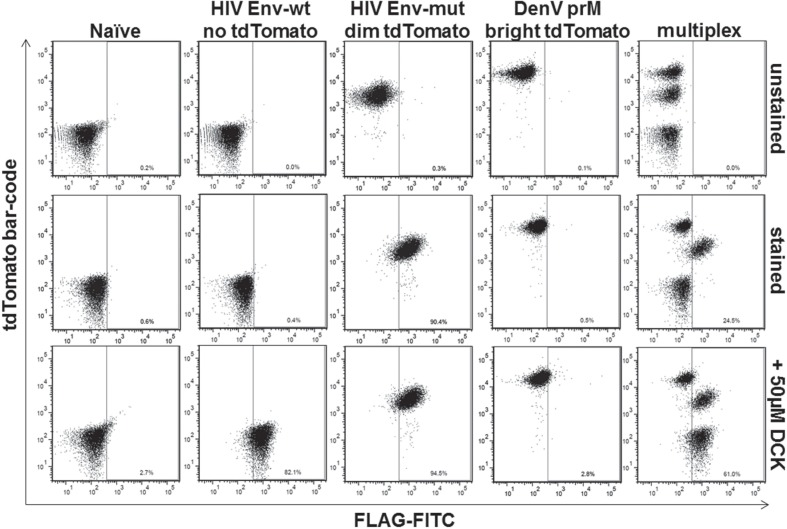
Establishment of the multiplexed assay in a 96-well format. Flow cytometry analysis of the fluorescent barcoded cell lines. Cell lines expressing HIV-1 Env-wt (no td Tomato), HIV-1 Env-mut (dim td Tomato), and DenV prM (bright td Tomato) were analyzed independently or in a mixed sample for multiplexing (last column) in a 96-well format. Analysis was performed with unstained cells, or cells stained with FLAG-FITC antibodies alone, or following treatment with the DCK Furin Inhibitor.

The multiplexed platform was then analyzed in a miniaturized setup to establish the conditions for HTS. For that purpose, a mixture containing equal amounts of each barcoded cell line was incubated with and without Furin DCK inhibitor in a 96-well plate format as previously described and analyzed for FLAG surface expression by flow cytometry. Each population was also analyzed independently for comparison. Within the mixture, FLAG positive populations were readily observed. Without inhibitor, the HIV Env-mut (dim td Tomato) was FLAG positive, whereas HIV Env-wt (negative td Tomato) and DenV prM (bright td Tomato) were negative, as expected (**Fig. 3**). On addition of DCK, HIV Env-wt cells reconstituted FLAG surface expression (**Fig. 3**). The DenV prM cells remained negative for FLAG, although we know that they express the HA epitope (**Fig. 1B** **and** **C**) and thus do display the cleaved scaffold on the surface. The DenV prM cells were incubated with higher concentrations of DCK but never recovered FLAG staining (data not shown). Although unexpected, these results hint at the possibility that an enzyme other than Furin is involved in the cleavage of DenV prM. Moreover, these results highlight the utility of multiplexing that can reveal differences between similar samples within the same well. The HIV Env-wt substrate can thus act as an internal control for specificity, because hits that block cleavage of one target but not the other can reveal specific competitors, as opposed to those that both block and target the general machinery.

## Pilot Screen of the Prestwick Chemical Library

**Figure 4.**
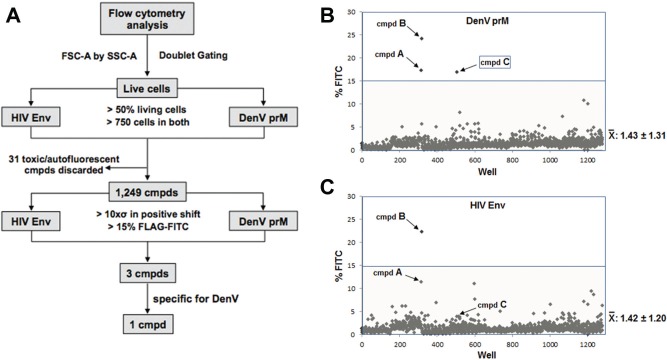
Pilot screen of the Prestwick Chemical Library. (**A**) Workflow of the screen and cutoff values for hits. The multiplexed assay consisting of a mixture of cells, expressing the DenV prM boundary assay (bright td Tomato cells) or the HIV-Env wt boundary assay (nonfluorescent cells), was screened in 96-well plates with one compound/well of the 1280 PCL compounds. (**B**) Results of the screen for DenV prM. (**C**) Results for HIV Env. Following staining with anti-FLAG-FITC and the stringent setting of cutoff parameters (refer to text for more details), three independent putative hits were found. The gray region indicates the <15% fluorescence cutoff. The overall average value of percentage FITC positive for the entire screen is indicated for each target. We have focused here on Compound C, a putative hit specific for DenV.

The calibrated-mulitplexed platform was ready to be screened against the PCL containing 1280 small molecules. Although our multiplexing technique based on fluorescent genetic barcoding can readily distinguish and separate three or more samples, it has not been tested in a screen before. Small molecules can affect cell fluorescence, which would undoubtedly interfere with the assay readout and hinder the screening process. The DenV prM bright td Tomato and the nonfluorescent HIV Env-wt cell line were used as a proof-of-principle for multiplexed screening. Briefly, multiplexed cells were incubated with the PCL at a 10 µM concentration in 1% DMSO solvent for 48 h and analyzed using the previously mentioned protocol. Autofluorescence and cytotoxicity were analyzed prior to FLAG staining to set the cutoff parameters required to determine FLAG positive hits in the subsequent analyses. The screen results are shown in **Figure 4**.

Briefly, live cells were defined based on forward scatter (FSC-A) and side scatter (SSC-A) profiles and subsequently gated for single cells by doublet gating (see **Fig. 4A** for a flowchart describing the screen and cutoff parameters for hit determination). Barcoded populations were then isolated by gating for td Tomato positive (DenV prM) or negative (HIV Env) cells, and only then analyzed for FLAG-FITC staining. Combined cutoff values for percentage of live cells (>50%) and number of events in each barcoded population (minimum 750) were established to ensure a more strict protocol and more reliable values. This eliminated 31 toxic/autofluorescent compounds from the data set. The remaining 1249 wells exhibited an average 1.43% ± 1.31 and 1.42% ± 1.20 FLAG-FITC signal for DenV prM and HIV Env cells, respectively, when treated with the PCL. Furthermore, each well contained a similar number of barcoded assay cells: 46.58% ± 4.20 td Tomato positive and 51.80% ± 4.08 td Tomato negative. Due to the robustness of the assay, initial hit cutoff values were established at an arbitrary 15% FLAG-FITC signal for both DenV (**Fig. 4B**) and HIV (**Fig. 4C**) assays. The 15% cutoff value was identified as at least a positive shift of 10 times the standard deviation (10×σ) over the average of all wells obtained in both DenV prM and HIV Env screens. With the establishment of these stringent cutoff values, both assays identified a combined number of three hits (Compounds A–C), establishing a hit rate of 0.23%.

To address any signal strength variation between plates, hits were reconfirmed to have a minimum of a 4×σ positive shift in the percentage of FLAG-FITC in comparison to the entire screen as well as to the respective individual plate. Furthermore, all hits had a minimum of 3.5×σ in MFI with respect to their individual plate. Due to the fact that unhealthy cells tend to exhibit higher background staining, FLAG positive populations were backgated for FSC-A and SSC-A to reanalyze possible cytotoxicity. In each of the hits, positive FLAG signal was identified only from the healthy cells as determined by FSC-A and SSC-A profiles.

**Figure 5.**
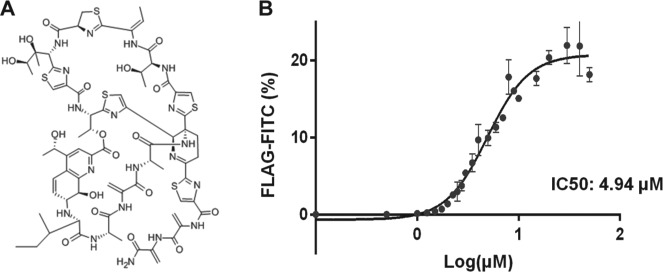
Dose response with thiostrepton. The putative DenV-specific hit obtained with Compound C (Thiostrepton) was cherry-picked for further analysis. (**A**) Chemical structure of Thiostrepton. (**B**) An independent source of dry material was used for calculating dose response and IC_50_. Percentage fluorescence following staining with anti-FLAG-FITC was analyzed by flow cytometry.

**Figure 6.**
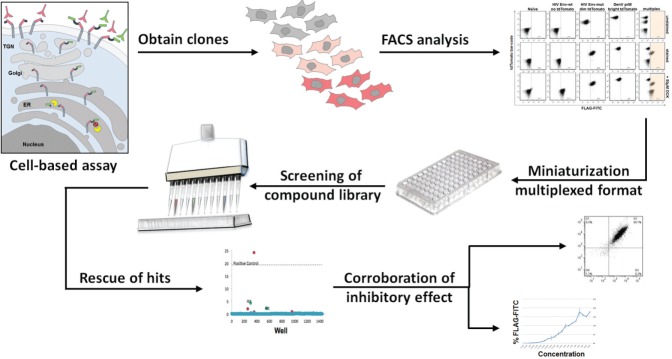
Model of the HTS approach undertaken in search for competitors of prM processing. Cells are engineered to carry the assay, as depicted in the leftmost panel. Cells carrying DenV prM and HIV Env-wt or HIV Env-mut as controls were engineered in genetically fluorescent barcoded cells to obtain three independent clones or cell lines. Flow cytometry analysis was performed with the independent cell lines and with the mixture for multiplexing. Miniaturization of the multiplexed assay with DenV prM and HIV Env-wt was performed and used to screen the PCL. Following rescue of putative hits based on stringent cutoff parameters, hits are reanalyzed by flow cytometry and for dose response.

As mentioned, the stringent and reliable cutoff values used to discard negative and false-positive hits revealed three putative hits referred to as A, B, and C. Compounds A and B affected both HIV Env and DenV prM processing, although the response in HIV Env for Compound A fell slightly lower than the 15% cutoff value. The screen identified one hit (Compound C) that was shown to be effective against DenV prM processing but not HIV Env. Compound C was revealed as Thiostrepton (**Fig. 5A**), a natural cyclic peptide from *Streptomyces* with antibiotic properties but no known activity against DenV.^35^ Thiostrepton exhibited a 6.7×σ positive shift in percentage FLAG staining in comparison to its local plate. To address potential bias from arbitrary gating, MFI was also analyzed. Thiostrepton also showed a significant shift in MFI, with a 9.3×σ positive shift. This analysis identified Thiostrepton as a clear outlier, indicating a putative ideal hit. In striking contrast, Thiostrepton did not result in a significant shift with the HIV Env wt boundary, as observed by only a 2×σ positive shift in both MFI and FLAG staining, much lower than the set cutoff values. Cherry-picking and dose response further corroborated Thiostrepton as a true positive hit, specifically targeting DenV prM processing. A nonlinear regression (*R*^2^ = .96) was used to determine that the IC_50_ had a value of 4.94 µM (**Fig. 5B**). The identification of dual-acting and specific inhibitors and competitors highlights the usability of the multiplexed screening platform. The pilot screening of the PCL using the cell-based platform and the rescue of at least one putative hit specifically targeting DenV prM demonstrated the utility of the assay for high-throughput drug discovery–based screens, the process of which is depicted in **Figure 6**.

## Discussion

We have presented for the first time a platform that couples multiplexing technologies with a robust live cell-based assay that relies on simple and straightforward antibody staining. In addition, the platform is the first of its kind to analyze competitors and inhibitors of DenV prM processing, an intriguing novel drug target against DenV infection. The assay monitors cleavage of DenV prM not only in a cellular context but also within the cellular compartment where prM processing occurs during DenV infection.

Here, we demonstrate the adaptation of our previously developed assay for the HIV Env protein to monitor DenV prM processing.^21^ The assay was readily adapted and represents a platform to study proteolysis of other substrates within the classical secretory pathway. Furthermore, the platform was miniaturized to a 96-well plate format, with no need of washes, and thus was easily adaptable to high-throughput screening, using highly calibrated small amounts of antibody to minimize nonspecific and background staining. It is worth mentioning that this adaptation likely relies on antibodies with high-affinity binding. To expand the capabilities of the assay and study the specificity of hits encountered during the screening process, we have multiplexed the assay using a previously described technique of fluorescent barcoding, further enhancing HTS capabilities.^24^ In this fashion, it is relatively easy to identify either broad-spectrum or specific targeted inhibitors/competitors. It is important to mention that although the assay is adapted for the monitoring of prM cleavage using 20 amino acids of the prM boundary, it is ultimately intended for the discovery of competitors of cleavage rather than inhibitors of the enzyme responsible for it, because inhibitors will most probably be toxic to the cell. The assay does not distinguish between competitors and inhibitors, and further characterization of hits is thus required to assess the nature of function and structure–activity relationships. Secondary screens will need to be performed with a larger prM boundary segment along with orthogonal cell-based assays.

To demonstrate the utility of the assay for drug screening, we performed a pilot screen of the PCL containing 1280 small molecules. The screen included DCK as a control drug against HIV-1 Env processing. The results obtained with DCK, known to inhibit Furin or Furin-like protein convertases, did not show similar results with the multiplexed cell lines. Whereas the HIV Env assay responded well to Furin or Furin-type convertase inhibition, as expected, the DenV prM assay did not. Importantly, the screen identified a hit, Thiostrepton, which was effective against DenV prM but not HIV Env processing. According to several reports in the literature, both DenV prM and HIV Env are recognized by the same protease, mainly Furin.^9,15,30^ However, the specificity and accuracy of our assay to monitor cleavage within the classical secretory pathway in living cells revealed that this might not be the case, and other host proteases within the secretory pathway could be involved. Further studies including knockdown experiments in the context of the assay may elucidate the specific proteases responsible for cleavage, as well as highlight the complexity of protease regulation within the secretory pathway. Conversely, it is possible that Thiostrepton serves as a competitor for cleavage, thus blocking DenV prM recognition and cleavage and not HIV Env. Although the assay cannot discriminate between inhibitors and competitors, it is the goal of the screen to find competitors, because inhibitors will most certainly have cytotoxic effects. Additional studies will be needed to establish the mechanism of action of Thiostrepton, and show whether it inhibits DenV infection, all required to confirm Thiostrepton as a hit compound for lead development. Thiostrepton, a cyclic peptide with antibiotic properties produced by several strains of the bacterial genus *Streptomyces*, has not been reported as a possible drug against DenV.

Although existing screening platforms have certainly led to the discovery of hits and lead compounds, many of these compounds fail in later phases of evaluation. To improve the success rate of hits and lead compound discovery, it is necessary to further improve both assays and diagnostics. Many current platforms use advantageous cell-based systems to provide a natural milieu for drug–target interaction, help identify the toxicity of compounds, and determine if compounds can enter the cell. Furthermore, multiplexing the assay against independent targets allows performing fewer screens and enables discrimination between broad mode-of-action inhibitors/competitors versus those specifically targeted. In addition, each independent screen performed in the multiplexed format can serve as an internal control for the other. Using these types of controls will facilitate the identification of real and relevant hits in high-throughput platforms. Our HTS platform represents the first of its kind that couples multiplexing, live-cell based analyses, and antibody-based detection of proteolytic processing within the secretory pathway using flow cytometry, yet it is easily adaptable to microscopy. Importantly, although the hit rate of our screen was established at the seemingly low 0.23%, it further demonstrates the specificity of the assay for monitoring cleavage in a very specific compartment of the cell. This, concomitantly with very stringent cutoff values and the inexistence of available drugs against HIV-1 Env and Denv prM processing, accounts for the low-success hit rate in our screen.

Because the assay monitors cleavage within the classical secretory pathway in living cells in a robust and reliable manner, it can be used in a more phenotypic-based approach screen. Conversely, because it can easily distinguish between cleavages of different substrates, probably by different enzymes, it can also be used as a target-based discovery tool. Compound C (Thiostrepton) proved beyond any doubt the usability of this new platform for HTS, performed here against DenV prM processing.

## References

[1] WHO. Dengue and Severe Dengue. http://www.who.int/mediacentre/factsheets/fs117/en/ (accessed Jan 6, 2014).

[2] Wilder-Smith A. Murray N. E. Quam M. Epidemiology of Dengue: Past, Present and Future Prospects. Clin. Epidemiol. 2013, 5, 299–309. 23990732 10.2147/CLEP.S34440 PMC3753061

[3] Bhatt S. Gething P. W. Brady O. J. The Global Distribution and Burden of Dengue. Nature. 2013, 496, 504–507. 23563266 10.1038/nature12060 PMC3651993

[4] Brady O. J. Gething P. W. Bhatt S. Refining the Global Spatial Limits of Dengue Virus Transmission by Evidence-Based Consensus. PLoS Negl. Trop. Dis. 2012, 6, e1760. 22880140 10.1371/journal.pntd.0001760 PMC3413714

[5] De La Guardia C. Lleonart R. Progress in the Identification of Dengue Virus Entry/Fusion Inhibitors. BioMed. Res. Int. 2014, 2014, e825039. 10.1155/2014/825039 PMC4135166 25157370

[6] Rodenhuis-Zybert I. A. Wilschut J. Smit J. M. Dengue Virus Life Cycle: Viral and Host Factors Modulating Infectivity. Cell. Mol. Life Sci. 2010, 67, 2773–2786. 20372965 10.1007/s00018-010-0357-z PMC11115823

[7] Kuhn R. J. Zhang W. Rossmann M. G. Structure of Dengue Virus: Implications for Flavivirus Organization, Maturation, and Fusion. Cell. 2002, 108, 717–725. 11893341 10.1016/s0092-8674(02)00660-8 PMC4152842

[8] Perera R. Kuhn R. J. Structural Proteomics of Dengue Virus. Curr. Opin. Microbiol. 2008, 11, 369–377. 18644250 10.1016/j.mib.2008.06.004 PMC2581888

[9] Junjhon J. Lausumpao M. Supasa S. Differential Modulation of prM Cleavage, Extracellular Particle Distribution, and Virus Infectivity by Conserved Residues at Nonfurin Consensus Positions of the Dengue Virus Pr-M Junction. J. Virol. 2008, 82, 10776–10791. 18715923 10.1128/JVI.01180-08 PMC2573171

[10] Keelapang P. Sriburi R. Supasa S. Alterations of Pr-M Cleavage and Virus Export in Pr-M Junction Chimeric Dengue Viruses. J. Virol. 2004, 78, 2367–2381. 14963133 10.1128/JVI.78.5.2367-2381.2004 PMC369205

[11] Che P. Tang H. Li Q. The Interaction between Claudin-1 and Dengue Viral prM/M Protein for Its Entry. Virology. 2013, 446, 303–313. 24074594 10.1016/j.virol.2013.08.009

[12] Wong S.-S. Haqshenas G. Gowans E. J. The Dengue Virus M Protein Localises to the Endoplasmic Reticulum and Forms Oligomers. FEBS Lett. 2012, 586, 1032–1037. 22569259 10.1016/j.febslet.2012.02.047

[13] Yu I.-M. Zhang W. Holdaway H. A. Structure of the Immature Dengue Virus at Low pH Primes Proteolytic Maturation. Science. 2008, 319, 1834–1837. 18369148 10.1126/science.1153264

[14] Tsai W.-Y. Hsieh S.-C. Lai C.-Y. C-Terminal Helical Domains of Dengue Virus Type 4 E Protein Affect the Expression/Stability of prM Protein and Conformation of prM and E Proteins. PLoS ONE. 2012, 7, e52600. 23300717 10.1371/journal.pone.0052600 PMC3530441

[15] Stadler K. Allison S. L. Schalich J. Heinz F. X. Proteolytic Activation of Tick-Borne Encephalitis Virus by Furin. J. Virol. 1997, 71, 8475–8481. 9343204 10.1128/jvi.71.11.8475-8481.1997 PMC192310

[16] Mukherjee S. Lin T.-Y. Dowd K. A. The Infectivity of prM-Containing Partially Mature West Nile Virus Does Not Require the Activity of Cellular Furin-like Proteases. J. Virol. 2011, 85, 12067–12072. 21880759 10.1128/JVI.05559-11 PMC3209279

[17] Wengler G. Wengler G. Cell-Associated West Nile Flavivirus Is Covered with E+pre-M Protein Heterodimers Which Are Destroyed and Reorganized by Proteolytic Cleavage during Virus Release. J. Virol. 1989, 63, 2521–2526. 2724410 10.1128/jvi.63.6.2521-2526.1989 PMC250716

[18] Zybert I. A. van der Ende-Metselaar H. Wilschut J. Functional Importance of Dengue Virus Maturation: Infectious Properties of Immature Virions. J. Gen. Virol. 2008, 89, 3047–3051. 19008392 10.1099/vir.0.2008/002535-0

[19] Rodenhuis-Zybert I. A. Wilschut J. Smit J. M. Partial Maturation: An Immune-Evasion Strategy of Dengue Virus? Trends Microbiol. 2011, 19, 248–254. 21388812 10.1016/j.tim.2011.02.002

[20] Martina B. E. E. Koraka P. Osterhaus A. D. M. E. Dengue Virus Pathogenesis: An Integrated View. Clin. Microbiol. Rev. 2009, 22, 564–581. 19822889 10.1128/CMR.00035-09 PMC2772360

[21] Stolp Z. D. Stotland A. Diaz S. A Novel Two-Tag System for Monitoring Transport and Cleavage through the Classical Secretory Pathway: Adaptation to HIV Envelope Processing. PloS ONE. 2013, 8, e68835. 23840860 10.1371/journal.pone.0068835 PMC3686725

[22] Curpan R. F. Simons P. C. Zhai D. High-Throughput Screen for the Chemical Inhibitors of Antiapoptotic Bcl-2 Family Proteins by Multiplex Flow Cytometry. Assay Drug Dev. Technol. 2011, 9, 465–474. 21561376 10.1089/adt.2010.0363 PMC3182036

[23] Saunders M. J. Graves S. W. Sklar L. A. High-Throughput Multiplex Flow Cytometry Screening for Botulinum Neurotoxin Type A Light Chain Protease Inhibitors. Assay Drug Dev. Technol. 2010, 8, 37–46. 20035615 10.1089/adt.2009.0219 PMC3096553

[24] Smurthwaite C. A. Hilton B. J. O’Hanlon R. Fluorescent Genetic Barcoding in Mammalian Cells for Enhanced Multiplexing Capabilities in Flow Cytometry. Cytometry A. 2014, 85, 105–113. 24700576 10.1002/cyto.a.22406

[25] Shum D. Smith J. L. Hirsch A. J. High-Content Assay to Identify Inhibitors of Dengue Virus Infection. ASSAY Drug Dev. Technol. 2010, 8, 553–570. 20973722 10.1089/adt.2010.0321 PMC2962577

[26] Scaturro P. Trist I. M. L. Paul D. Characterization of the Mode of Action of a Potent Dengue Virus Capsid Inhibitor. J. Virol. 2014, 88, 11540–11555. 25056895 10.1128/JVI.01745-14 PMC4178822

[27] Rothan H. A. Zulqarnain M. Ammar Y. A. Screening of Antiviral Activities in Medicinal Plants Extracts against Dengue Virus Using Dengue NS2B-NS3 Protease Assay. Trop. Biomed. 2014, 31, 286–296. 25134897

[28] Checkley M. A. Luttge B. G. Freed E. O. HIV-1 Envelope Glycoprotein Biosynthesis, Trafficking, and Incorporation. J. Mol. Biol. 2011, 410, 582–608. 21762802 10.1016/j.jmb.2011.04.042 PMC3139147

[29] Molloy S. S. Thomas L. VanSlyke J. K. Intracellular Trafficking and Activation of the Furin Proprotein Convertase: Localization to the TGN and Recycling from the Cell Surface. EMBO J. 1994, 13, 18–33. 7508380 10.1002/j.1460-2075.1994.tb06231.x PMC394775

[30] Tian S. Huang Q. Fang Y. FurinDB: A Database of 20-Residue Furin Cleavage Site Motifs, Substrates and Their Associated Drugs. Int. J. Mol. Sci. 2011, 12, 1060–1065. 21541042 10.3390/ijms12021060 PMC3083689

[31] Molloy S. S. Bresnahan P. A. Leppla S. H. Human Furin Is a Calcium-Dependent Serine Endoprotease That Recognizes the Sequence Arg-X-X-Arg and Efficiently Cleaves Anthrax Toxin Protective Antigen. J. Biol. Chem. 1992, 267, 16396–16402. 1644824

[32] Henrich S. Cameron A. Bourenkov G. P. The Crystal Structure of the Proprotein Processing Proteinase Furin Explains Its Stringent Specificity. Nat. Struct. Biol. 2003, 10, 520–526. 12794637 10.1038/nsb941

[33] Decroly E. Benjannet S. Savaria D. Comparative Functional Role of PC7 and Furin in the Processing of the HIV Envelope Glycoprotein gp160. FEBS Lett. 1997, 405, 68–72. 9094426 10.1016/s0014-5793(97)00156-7

[34] Marchette N. J. Halstead S. B. Falkler W. A. Studies on the Pathogenesis of Dengue Infection in Monkeys. III. Sequential Distribution of Virus in Primary and Heterologous Infections. J. Infect. Dis. 1973, 128, 23–30. 4198025 10.1093/infdis/128.1.23

[35] Bond C. S. Shaw M. P. Alphey M. S. Structure of the Macrocycle Thiostrepton Solved Using the Anomalous Dispersion Contribution of Sulfur. Acta Crystallogr. Sect. D. 2001, 57, 755–758. 11320328 10.1107/s0907444901003134

